# Supplementary material for: 3D-Printed Bioactive Scaffold Loaded with GW9508 Promotes Critical-Size Bone Defect Repair by Regulating Intracellular Metabolism
Source: Bioengineering (Basel). 2023 Apr 27;10(5):535. doi: 10.3390/bioengineering10050535 (PMC10215968; doi:10.3390/bioengineering10050535)
Supplement: Supplementary file 1 [file bioengineering-10-00535-s001.zip › bioengineering-2230875-supplementary.pdf]

bioengineering

MDPI

Article

3D-Printed Bioactive Scaffold Loaded with GW9508 Promotes Critical-Size Bone Defect Repair by Regulating Intracellular Metabolism

Fangli Huang [1,†], Xiao Liu [1,†], Xihong Fu [2,†], Yan Chen [1], Dong Jiang [2], Tingxuan Wang [1], Rongcheng Hu [2], Xuenong Zou 1, Hao Hu [1,*] and Chun Liu [1,2,*]

[1] Department of Spine Surgery and Guangdong Provincial Key Laboratory of Orthopaedics and Traumatology, Sun Yat-sen University First Affiliated Hospital, Guangzhou 51080, China; huangfli@mail2.sysu.edu.cn (F.H.); liux539@mail.sysu.edu.cn (X.L.); cheny585@mail2.sysu.edu.cn (Y.C.); wangtx28@mail2.sysu.edu.cn (T.W.); zouxuen@mail.sysu.edu.cn (X.Z.)

[2] Precision Medicine Institute, Sun Yat-sen University First Affiliated Hospital, Guangzhou 51080, China; fuxh7@mail2.sysu.edu.cn (X.F.);jiangd28@mail2.sysu.edu.cn (D.J.);hurch5@mail2.sysu.edu.cn

* Correspondence: huhao7@mail2.sysu.edu.cn (H.H.); liuch393@mail.sysu.edu.cn (C.L.)

† These authors contributed equally to this work and share first authorship.

Supplementary Materials

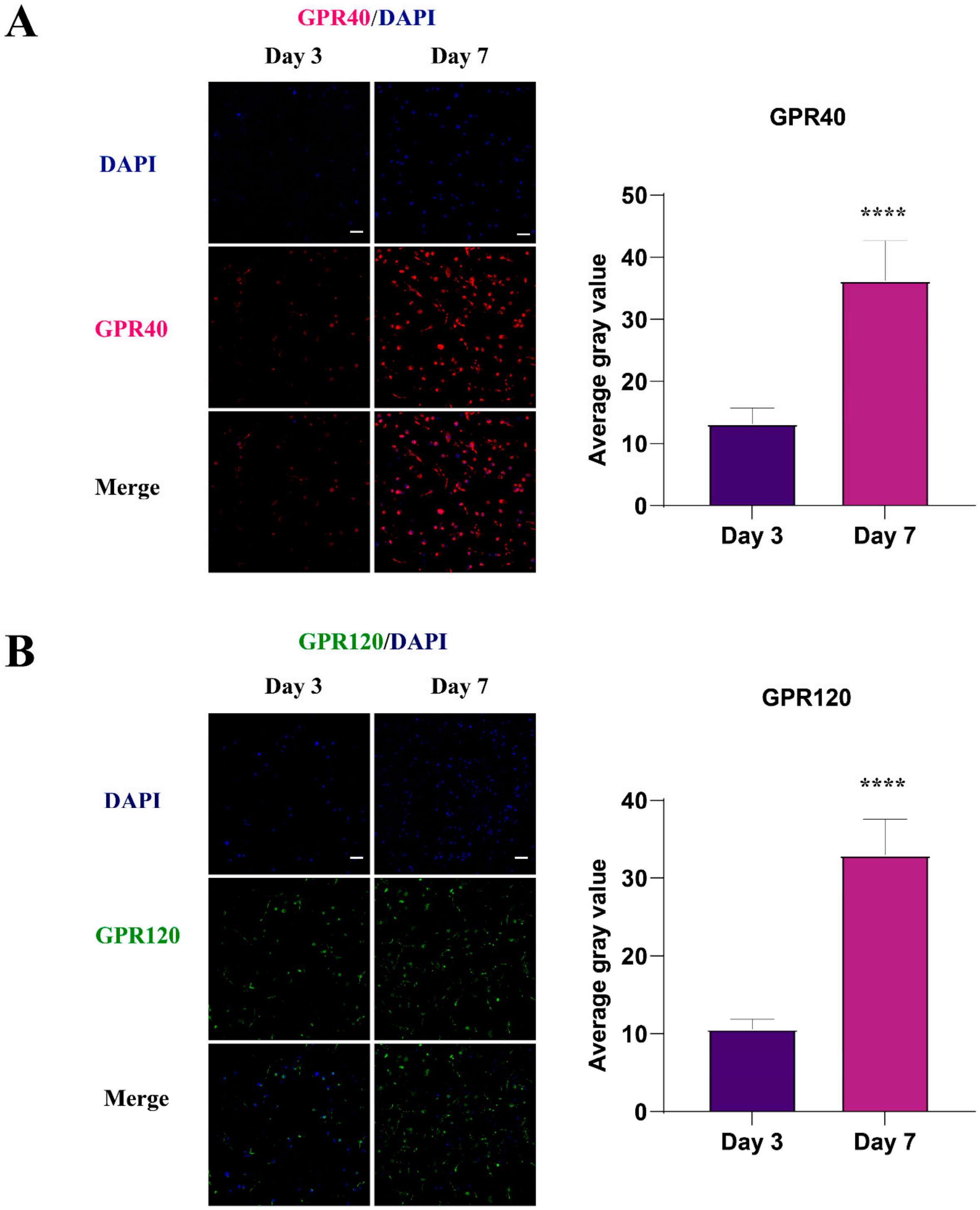

Figure S1. Immunofluorescence staining of (A) GPR 40 (Red) and (B) GPR 120 (Green) on rat BMSCs cultivated in COLI/HA/Alg hydrogel at Day3 and Day7. Scale bar = 50 μm. Significance was determined using one-way analysis of variance test. n=6, "ns" represents no significant difference,****P < 0.0001.

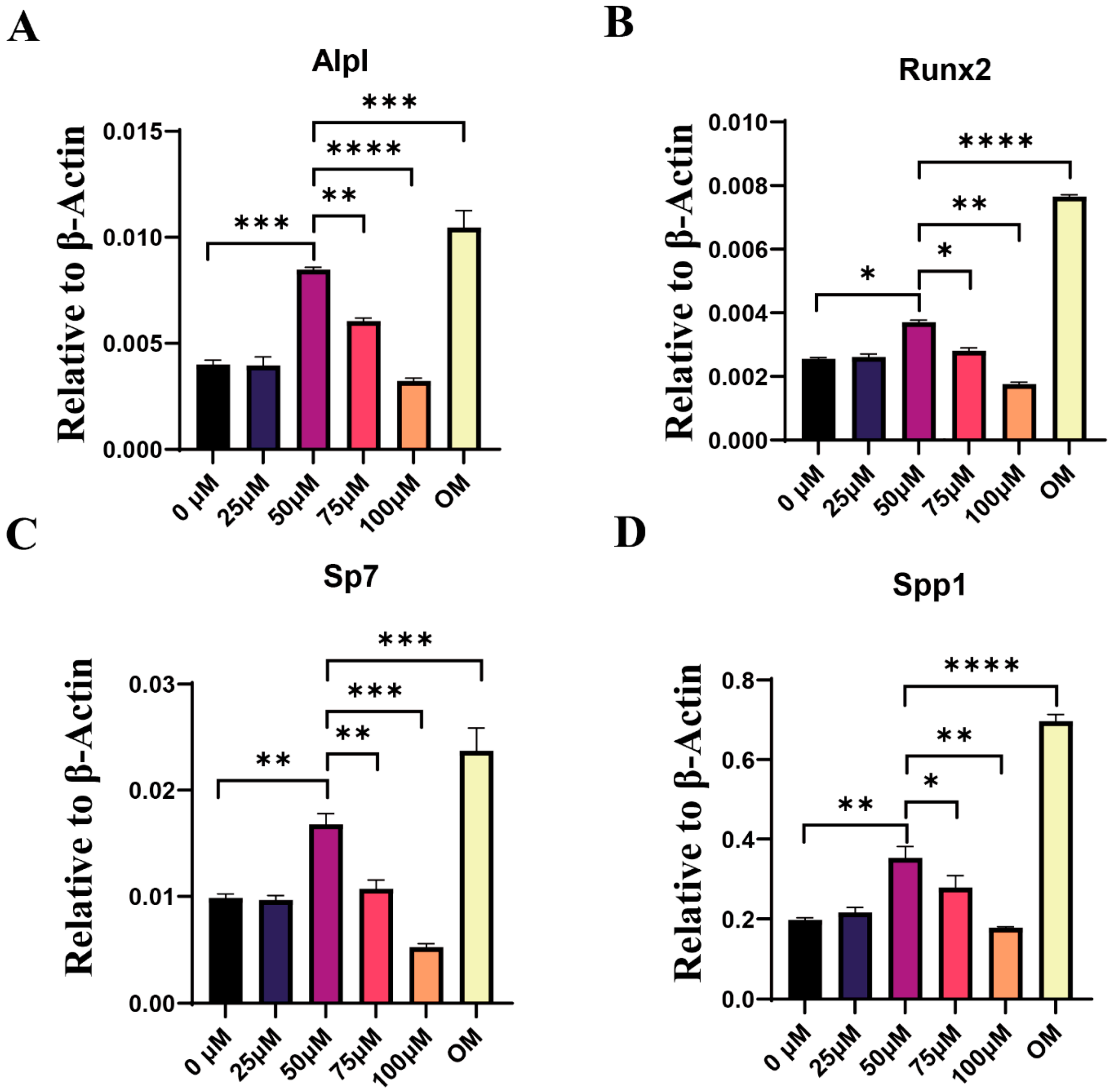

Figure S2. Effects of GW9508 on the osteogenic differentiation of rat Bone marrow mesenchymal stem cells (using β-actin as reference gene) relative to β-actin. A-D) Quantitative analysis of the gene expression of Alp (A), Runx2 (B), Sp7 (C), and Spp1 (D) in BMSCs at Day 14 relative to β-actin. Significance was determined using one-way analysis of variance test. n=4, "ns"represents no significant difference,*P < 0.05; **P < 0.01; ***P < 0.001; ****P < 0.0001.
